# *Leptospira* in Working Horses From Rural Ecuador: A Neglected Occupational Risk

**DOI:** 10.1155/jotm/8883896

**Published:** 2026-06-23

**Authors:** Eduardo A. Díaz, Carolina Sáenz, Diego Guzmán, Juan S. Galecio, Talima Pearson, Verónica Barragán

**Affiliations:** ^1^ Escuela de Medicina Veterinaria, Colegio de Ciencias de la Salud, Universidad San Francisco de Quito, Cumbayá, Quito, Ecuador, usfq.edu.ec; ^2^ Fundación GAIAS Europa de la Comunitat Valenciana, Universidad San Francisco de Quito, Valencia, Spain, usfq.edu.ec; ^3^ Hospital de Fauna Silvestre Tueri, Instituto iBIOTROP, Universidad San Francisco de Quito, Cumbayá, Quito, Ecuador, usfq.edu.ec; ^4^ Instituto de Microbiología, Colegio de Ciencias Biológicas y Ambientales, Universidad San Francisco de Quito, Cumbayá, Quito, Ecuador, usfq.edu.ec; ^5^ Pathogen and Microbiome Institute, Northern Arizona University, Flagstaff, Arizona, USA, nau.edu

**Keywords:** equine, MAT, One Health, PCR, seroconversion, tropical disease, zoonosis

## Abstract

Neglected tropical zoonotic diseases such as leptospirosis continue to have a significant impact on animal and human health, especially in low‐income countries due to limitations in surveillance and knowledge gaps to guide mitigation. In this context, equines have the potential to play an important role in the maintenance and transmission of *Leptospira* spp. due to their susceptibility to a variety of serovars (often without signs of disease) and close contact with humans. To better understand the epidemiological role of horses, we evaluated exposure and identified circulating leptospires by collecting blood and urine samples from 13 horses used on agricultural and livestock work in the rural areas of La Concordia and Mompiche, located near the coast of Ecuador. Microscopic agglutination tests showed anti‐*leptospira* antibodies for a variety of serotypes in 100% of 9 serum samples analyzed and pathogenic *Leptospira* DNA from *L. interrogans* and *L. santarosai* in 61.5% of 13 urine samples. These results indicate that exposure of horses to *Leptospira* is common, and a substantial proportion may actively excrete the pathogen without showing signs of disease. While the small sample size and single time point limit definitive conclusions, in areas where horses interact closely with humans and other animals, these findings suggest that horses could play a role in local environmental cycling of *Leptospira*. Our findings emphasize the need to strengthen preventive measures in both veterinary and public health areas based on hygiene and sanitary practices that are aligned with a One Health approach.

## 1. Introduction

Leptospirosis disproportionately affects individuals living in impoverished and resource‐limited settings, particularly in low‐income countries where inadequate sanitation and lack of routine surveillance contribute to its burden [1]. Humans contract leptospirosis through direct contact with urine from infected animals or indirect contact with urine‐contaminated environments [2]. Although infected people may be asymptomatic or mildly symptomatic, leptospirosis is estimated to cause more than 1 million severe cases and almost 60,000 deaths each year worldwide [3]. The epidemiological patterns of leptospirosis are closely related to the climate, with heavy rains, floods, and high temperatures in tropical countries being important risk factors that contribute to the persistence of leptospires in the environment [4]. Once the leptospires are shed and persist in the environment, a wide variety of animals could become infected. In humans, the risk of contracting the disease increases in people with peridomestic animals and those who work with animals, as both direct transmission from the infected host and indirect from the host’s contaminated environment can occur [5].

Working equines, which support agriculture and livestock production in rural communities in low‐ and middle‐income countries, provide a crucial contribution to achieving the Sustainable Development Goals of the 2030 Agenda [6]. However, they are often neglected in public health policies, despite the potential risks of emerging infectious disease transmission [7, 8]. The incidence of leptospirosis in horses remains uncertain due to a lack of standardization across studies, but recent publications have shown that equine leptospirosis may be more common than expected in Latin America [9, 10]. In Ecuador, the equine population has increased considerably, especially in rural areas where horses continue to be essential for work on agricultural and livestock farms [11]. As in many other tropical countries, bioclimatic conditions contribute to leptospirosis endemicity, especially in rural areas, where inadequate hygienic practices and sanitary infrastructure have resulted in an increase in human cases [12, 13]. In rural Ecuador, prevalence in peridomestic rodents is low, but prevalence of *Leptospira* spp. and leptospirosis in peridomestic cattle and pigs is high and extremely high in dogs. These patterns suggest a complex epidemiological landscape where peridomestic livestock play important roles in *Leptospira* cycling and risk to humans that is different from urban environments [14–17], but research on the role of horses in local epidemiology is lacking.

Given their potential to interact with multiple host species, their susceptibility to a variety of serotypes that are likely to cause disease in other species, and their ability to shed large amounts of leptospires into the environment, horses may act as sentinel species for *Leptospira* monitoring [18]. However, data on the role of horses in leptospirosis epidemiology in South America remain limited. Therefore, the objective of this study was to build knowledge about the potential epidemiological roles of horses in Ecuador by assessing exposure to *Leptospira* spp. and identifying circulating serogroups and species by collecting and analyzing blood and urine samples from working horses in rural areas.

## 2. Materials and Methods

### 2.1. Ethics Statement

The study involved blood and urine samples obtained from horses for diagnostic purposes by certified veterinarians. Owners consented to animal sampling and use of all associated data for the study. Sampling protocols were approved by the Ethics Committee on the Use of Animals in Research and Teaching–USFQ: 2023‐004.

### 2.2. Study Site

The horse samples were collected from two different rural farms in Ecuador that are about 185 km (115 miles) apart from each other. The first was in the province of Santo Domingo, at La Concordia (Latitude: −0.0139,225, Longitude: −79.3793327), and the second in the province of Esmeraldas, at Mompiche (Latitude: 0.4921267°N; Longitude: 80.0403,202°W). Mompiche is characterized primarily by degraded landscapes with limited primary forest and a predominance of domestic animal populations. In contrast, La Concordia includes areas of primary forest that support native wildlife.

### 2.3. Sampled Population

A total of 13 horses were sampled for this study. Samples were collected from seven crossbred horses from a farm in the town of La Concordia on February 24^th^, 2024 and from a farm in the town of Mompiche on December 21^st^, 2024. These farms were selected as each contained several horses but differed in location and potential to interact with other species in the immediate environment. All individual horses present during the sampling dates were included. From La Concordia, the sampled horses include three males and four females, all between five and seven years of age. The six crossbred horses sampled from Mompiche included five males and one female, between the ages of five and seven years. Specifically, the animals included in this study were working horses primarily used for agricultural labor and transportation. All animals were owner‐managed and maintained under extensive or semiextensive conditions. Housing infrastructure was limited, and water was obtained from natural or communal sources. Feeding practices consisted mainly of natural pasture and agricultural by‐products, with little or no nutritional supplementation. Access to veterinary care was limited, and preventive health measures such as vaccination and deworming were absent or irregular at the time of sampling. Neither the owner nor the workers reported signs or a history of leptospirosis in any of the horses; veterinarians also found no signs of disease at the time of sample collection, and there was no known leptospirosis outbreak in any species at the time. Owners reported that none of the animals had been vaccinated against leptospirosis.

Urine samples were collected during spontaneous micturition following the intravenous administration of a single dose of furosemide (0.5 mg/kg). Samples were obtained in sterile containers, and to preserve nucleic acid integrity, 4 mL of urine was immediately mixed with 4 mL of 2× DNA/RNA Shield (Zymo Research, USA) in sterile 10 mL tubes. To minimize animal stress and avoid interference with urine voiding, blood sampling was performed after urine collection. Blood samples were obtained by jugular venipuncture into 10 mL tubes without anticoagulant. Serum was separated by centrifugation at 5000 rpm for 5 min and subsequently aliquoted into sterile 2 mL tubes. All serum and urine samples were placed in insulated containers with ice packs and transported to the Institute of Microbiology at Universidad San Francisco de Quito (USFQ). Upon arrival, samples were stored at −20°C within 24 h of collection until further analysis.

### 2.4. Serological Analysis

Serum samples were analyzed at the National Reference Laboratory for Animal Diagnostics in Quito, Ecuador (AGROCALIDAD) by microscopic agglutination test (MAT) using standard methods [19] and performed with a panel of 23 serovars from *Leptospira interrogans*: serogroup Icterohaemorrhagiae (serovar Icterohaemorrhagiae and Copenhageni), serogroup Canicola (serovar Canicola), serogroup Australis (serovar Australis and Bratislava), serogroup Autumnalis (serovar Autumnalis), Bataviae (serovar Bataviae), Pyrogenes (serovar Pyrogenes and Borincana), Ballum (serovar Castellonis), Celledoni (serovar Celledoni), Djasiman (serovar Djasiman), Grippotyphosa (serovar Grippotyphosa), Sejroe (serovar Hardjo, Saxkooebing, Wolffi, and Sejroe), Hebdomadis (serovar Hebdomadis), Javanica (serovar Javanica), Panama (serovar Panama), Pomona (serovar Pomona), Shermani (serovar Shermani), and Tarassovi (serovar Tarassovi). MAT results were visualized by dark field microscopy, and the final titer was assigned as the serum dilution that promotes 50% agglutination. Samples with titers ≥ 1:100 were considered positive for antileptospiral antibodies. The serogroup with the highest titer was recorded for samples that reacted with more than one serogroup; samples were labeled ‘cross‐reactive’ if the highest titer value was found across multiple serogroups.

### 2.5. *Leptospira* DNA Detection and Identification

For molecular detection of pathogenic *Leptospira* spp. in horse urine, DNA was extracted using the DNeasy Blood and Tissue kit (Qiagen, CA, USA). DNA was tested using two previously described TaqMan assays specific for the pathogenic *Leptospira* clade: one assay targets the lipL32 gene, and the other targets a SNP in the 16SrRNA gene [14, 20]. As described in Guzman et al. [16], a sample was considered positive when at least one of the assays detected leptospiral DNA. *Leptospira* identification was performed by sequencing a secY gene fragment as described in Mosquera et al. [21].

**FIGURE 1:**
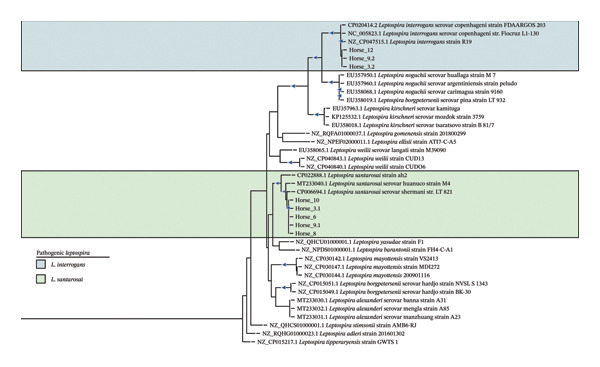
Phylogenetic relationships of the sequenced samples. Bootstrap support for nodes appearing in > 85% of 500 iterations is indicated by a blue triangle. Sequenced samples from this project are indicated with bolded text. In Samples 3 and 9, two species of *Leptospira* were detected (3.1, 3.2, 9.1, and 9.2). The tree was rooted using a sequence from *Leptonema illini* DSM 21528.

Consensus sequences were obtained using the Amplicon_sorter pipeline (v2024‐10‐16) without using any external references [22]. This pipeline can generate multiple consensus sequences that may vary in similarity and length. The resulting sequences were then compared with representative sequences of each *Leptospira* species available from GenBank. A phylogenetic tree was constructed in MEGA11 [23] using the Neighbor‐Joining method [24], along with the Maximum Composite Likelihood model and 500 bootstrap replicates, followed by phylogenetic tree visualization using iTOL (Figure 1).

All raw sequencing reads were deposited in the NCBI Sequence Read Archive (SRA) under BioprojectPRJNA1226566, with SRA accession numbers SRX27755983 and SRX27755990.

## 3. Results

Anti‐*leptospira* antibodies with titers ≥ 1:100 were detected in 100% of analyzed sera (blood samples from four horses were not available, as the animals had been relocated for work‐related activities following urine collection). Five *Leptospira* serogroups were identified in six horses: two animals showed agglutination with Panama, one with Pomona, one with Hebdomadis, one with Canicola, and one with Grippotyphosa; three horses showed cross‐reactions with multiple serogroups. The Canicola serogroup presented the highest titer (1:3200), followed by Hebdomadis (1:1600), Panama (1:800), Grippotyphosa (1:400), and Pomona (1:100) (Table 1).

**TABLE 1:** *Leptospira* serogroups and pathogenic *Leptospira* DNA identified in working horses from La Concordia and Mompiche localities.

Horse	Sex	Locality	*Leptospira* serogroup	*Leptospira* DNA
H1	M	La Concordia	Pomona 1:100	Not detected

H2	M	La Concordia	Panama 1:400	Not detected

H3	M	La Concordia	Cross‐reactive (Canicola, Panama, Pomona, Pyrogenes 1:200)	*L. santarosai* *L. interrogans*

H4	M	La Concordia	Cross‐reactive (Bataviae, Hebdomadis 1:200)	Not detected

H5	F	La Concordia	Panama 1:800	Not detected

H6	F	La Concordia	Hebdomadis 1:1600	*L. santarosai*

H7	F	La Concordia	Canicola 1:3200	Not detected

H8	M	Mompiche	Not available	*L. santarosai*

H9	M	Mompiche	Not available	*L. santarosai* *L. interrogans*

H10	M	Mompiche	Cross‐reactive (Autumnalis 1:100 Celledonis 1:100)	*L. santarosai*

H11	M	Mompiche	Not available	Not detected

H12	M	Mompiche	Grippotyphosa 1:400	*L. interrogans*

H13	F	Mompiche	Not available	Not detected

*Note:* M = male; F = female; NA = blood sample not available; ND = *Leptospira* DNA not detected; Cross‐reactive = multiple serogroups with highest titer. Not available = horses that were relocated for work‐related activities after urine collection and from whom blood samples could not be obtained. Not detected = samples that were tested for molecular detection of pathogenic *Leptospira* spp. but yielded no detectable leptospiral DNA.

*Leptospira* DNA was detected in three out of seven horse urine samples (42.8%) in La Concordia and five out of six (83.3%) in animals from Mompiche. DNA sequencing of the secY gene fragment allowed us to identify *Leptospira santarosai and L. interrogans*. Coinfection with two different species of pathogenic *Leptospira* was detected in one horse from each locality (Table 1).

## 4. Discussion

The seropositivity recorded in 100% of the analyzed horse sera indicates exposure to multiple serogroups and suggests frequent exposure to *Leptospira* spp. These results are consistent with high prevalence among peridomestic livestock in Ecuador and previous reports on leptospirosis in horses [14–16, 18, 20, 25, 26], suggesting that horse leptospirosis is likely endemic and very common in this region. Interestingly, the predominant serogroups vary between studies, with the highest MAT titers observed for the Canicola (1:3200), Hebdomadis (1:1600), and Panama (1:800) serogroups, all detected in horses from La Concordia. This variation may reflect temporal patterns, high diversity in circulating serotypes, or ecological differences in study sites. For example, Mompiche lacks primary forest and is characterized mainly by domestic animal populations, whereas La Concordia includes areas of primary forest that support native wildlife. The Canicola, Hebdomadis, and Panama serogroup have previously been reported in Ecuador across a wide range of hosts, including wildlife, livestock, domestic animals, and humans [20–34], suggesting potential environmental and multihost transmission pathways at the animal–human interface. These findings align with the broader epidemiological context in Ecuador, where leptospirosis is endemic across multiple regions and both human and animal populations are frequently exposed to diverse *Leptospira* serogroups [13].

Between 2022 and 2024, 16 cases of human leptospirosis were confirmed in the parish where Mompiche is located. During the same period, 246 cases were reported in the parish of La Concordia, according to the Ministry of Public Health of Ecuador (MSP‐MSP‐2025‐1035‐O), demonstrating the active presence of this zoonosis in the area and the need for ongoing preventive sanitary control measures and epidemiological surveillance. Our results provide support for using horses as sentinel species [18], although additional sample collection from a variety of animals and regions needs to be done to establish the extent to which samples from horses are representative of the load and types of circulating leptospires and risk to humans. Unfortunately, although the MAT remains the gold standard for diagnosis, previous studies have relied on single‐sample analyses, which provide information on past exposure [35], but not on current infection status. There is an urgent need for studies that correlate clinical signs with the detection of *Leptospira* DNA in animal urine. This would be valuable not only for improving the accuracy of leptospirosis diagnosis but also to understand which strains cause acute disease, which are carried for longer periods of time (host‐adapted), which animals are important reservoir hosts [36], and whether certain animals can be used as sentinels for monitoring circulating pathogens and risk.

Infecting strains will influence disease manifestations, shedding patterns, and risk to other species. While horses can develop acute clinical disease, the majority of infected horses are asymptomatic carriers, potentially shedding leptospires in their urine for extended periods [37]. However, leptospiruria can be intermittent, and monitoring strategies should incorporate serial urine testing to obtain more sensitive results [38, 39]. We detected *Leptospira* DNA in the urine of 61.5% of apparently healthy horses, although these data undoubtedly underestimate prevalence, as only one urine sample per animal was analyzed. Sequence analysis identified *Leptospira interrogans* and *Leptospira santarosai*; notably, mixed carriage was detected in two horses (H3 from La Concordia and H9 from Mompiche). Both *L. interrogans* and *L. santarosai* have been documented in human infections and in domestic and synanthropic animals, including cattle, pigs, and rats, in Ecuador [14, 16]. Their identification in working equines highlights the potential epidemiological interface between animal reservoirs and human transmission cycles. Moreover, the detection of mixed carriage underscores the potential role of horses as sentinels of locally circulating *Leptospira* species and supports their possible contribution to pathogen maintenance and environmental dissemination through urinary shedding. Currently, the Ministry of Agriculture and Livestock does not include leptospirosis among the notifiable diseases, nor does it recommend anti‐*leptospira* vaccination in the National Equine Health Program [11]. Implementing vaccination may reduce the risk to other species that interact with these horses.

A limitation of this study is that sampling was restricted to working horses from only two farms, and only a single specimen was obtained from each animal, precluding longitudinal assessment of temporal variation in bacterial shedding or serological status. Owing to sociopolitical instability in Ecuador [40], it was not feasible to perform repeat sampling or to collect additional samples from these or other equines in the same region. Moreover, the USFQ continues to suspend field research activities in coastal areas due to persistent security and logistical constraints, which has prevented continuation of the study in this region.

An additional limitation relates to the interpretation of MAT results. While MAT provides evidence of exposure to *Leptospira* spp., it does not reliably distinguish between past and current infection when based on a single serum sample. The absence of paired sera precluded assessment of seroconversion or a ≥ 4‐fold rise in antibody titers, which are standard approaches to infer recent or active infection. Similarly, reliance on a single urine sample per animal may have reduced sensitivity for detecting leptospiral shedding due to the intermittent nature of leptospiruria. Serial urine sampling over multiple timepoints would improve detection of active shedding.

Finally, the general lack of specificity of MAT means that these results may not be indicative of the infecting serovars or serogroups, making it difficult to compare circulating serotypes across studies. In Ecuador, efforts to isolate, culture, preserve, and antigenically characterize autochthonous *Leptospira* strains remain limited. Consequently, National Reference Laboratories typically perform MAT using panels composed predominantly of non‐local *Leptospira interrogans* serovars [13].

Nevertheless, the results obtained add new information to the little‐known epidemiology of equine leptospirosis in rural, low‐income areas [18]. Our findings indicate that working horses may act as asymptomatic carriers that excrete *Leptospira*, suggesting the potential for transmission to humans and other animals. However, given the small sample size and single time point per animal, these findings should be interpreted with caution. These data, coupled with our previous work that shows a high prevalence in peridomestic livestock and dogs where human–animal interactions are common [14, 16], emphasize the need for public health entities to coordinate with the veterinary sector to implement longitudinal surveillance of human leptospirosis outbreaks related to horses and other animals. *Leptospira* has been frequently found at the human–animal–environment interface, but there is still much to learn about the epidemiology of leptospirosis in diverse ecosystems around the world in order to develop effective mitigation strategies.

## 5. Conclusions

In Ecuador, leptospirosis remains a re‐emerging zoonosis of public health concern. Equines, which are integral to the livelihoods of low‐income communities, are frequently overlooked in national public health strategies. Our findings suggest that working horses, similar to peridomestic livestock, may play a role in the local transmission dynamics of *Leptospira* spp. although the small sample size and single time point per animal limit definitive conclusions regarding reservoir status or direct transmission to humans. Future surveillance‐based investigations should adopt a One Health framework, incorporating domestic animals, wildlife, humans, and environmental sources to better define the occupational and ecological risks associated with equine leptospirosis. Concurrently, comprehensive nationwide epidemiological surveys are needed to elucidate transmission patterns at the population level. Priorities should include expanded molecular genotyping of circulating strains, strengthened laboratory diagnostic capacity, and improved access to standardized surveillance data. Although the combined use of MAT for screening, followed by PCR and amplicon sequencing, proved adequate for identifying carrier animals, the isolation and characterization of locally circulating serovars are essential to improve diagnostic accuracy and inform regionally appropriate vaccination strategies. Standard approaches to better characterize infection dynamics, such as paired sera and serial urine sampling, should be prioritized in future research. Until such advances are achieved, the implementation of reinforced hygienic–sanitary measures in both public and veterinary health sectors is strongly recommended to enhance prevention and control efforts.

## Author Contributions

Conceptualization: Eduardo A. Díaz and Verónica Barragán; methodology: Eduardo A. Díaz, Carolina Sáenz, Diego Guzmán, and Juan S. Galecio; validation: Eduardo A. Díaz and Verónica Barragán; formal analysis: Eduardo A. Díaz, Diego Guzmán, and Verónica Barragán; investigation: Eduardo A. Díaz; resources: Verónica Barragán; data curation: Eduardo A. Díaz; writing–original draft preparation: Eduardo A. Díaz; writing–review and editing: Eduardo A. Díaz, Carolina Sáenz, Diego Guzmán, Juan S. Galecio, Talima Pearson, and Verónica Barragán; funding acquisition: Talima Pearson and Verónica Barragán.

## Funding

This research was carried out thanks to the National Institute of Allergy and Infectious Diseases in the National Institutes of Health (NIH/NIAID) award number R01AI172924 (Pearson).

## Disclosure

All authors have read and agreed to the published version of the manuscript.

## Ethics Statement

All sample collections were carried out with the informed consent of the owners, who also authorized the use of associated data for research purposes. The sampling procedures were performed by certified veterinarians for diagnostic purposes and approved by the Ethics Committee on the Use of Animals in Research and Teaching at USFQ (Protocol No. 2023‐004).

## Conflicts of Interest

The authors declare no conflicts of interest.

## Data Availability

The data that support the findings of this study are openly available in NCBI Sequence Read Archive at https://www.ncbi.nlm.nih.gov/bioproject/PRJNA1226566/, reference number BioprojectPRJNA1226566.
